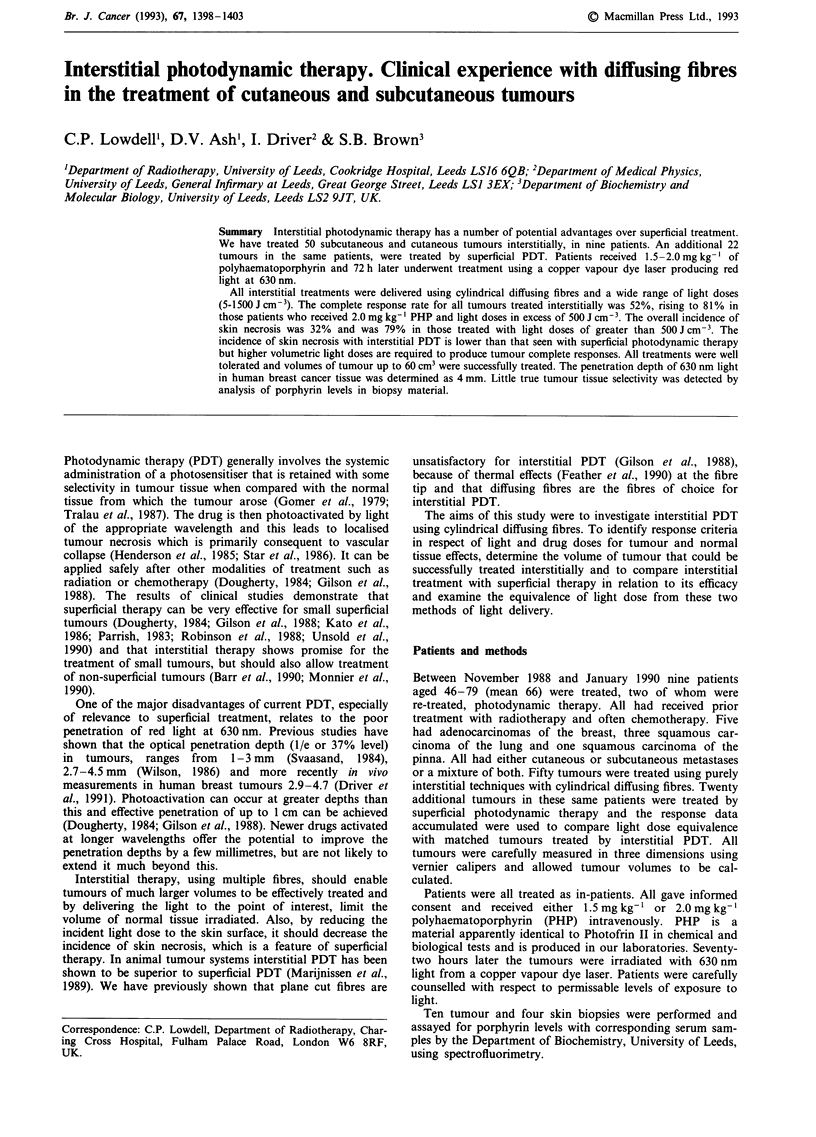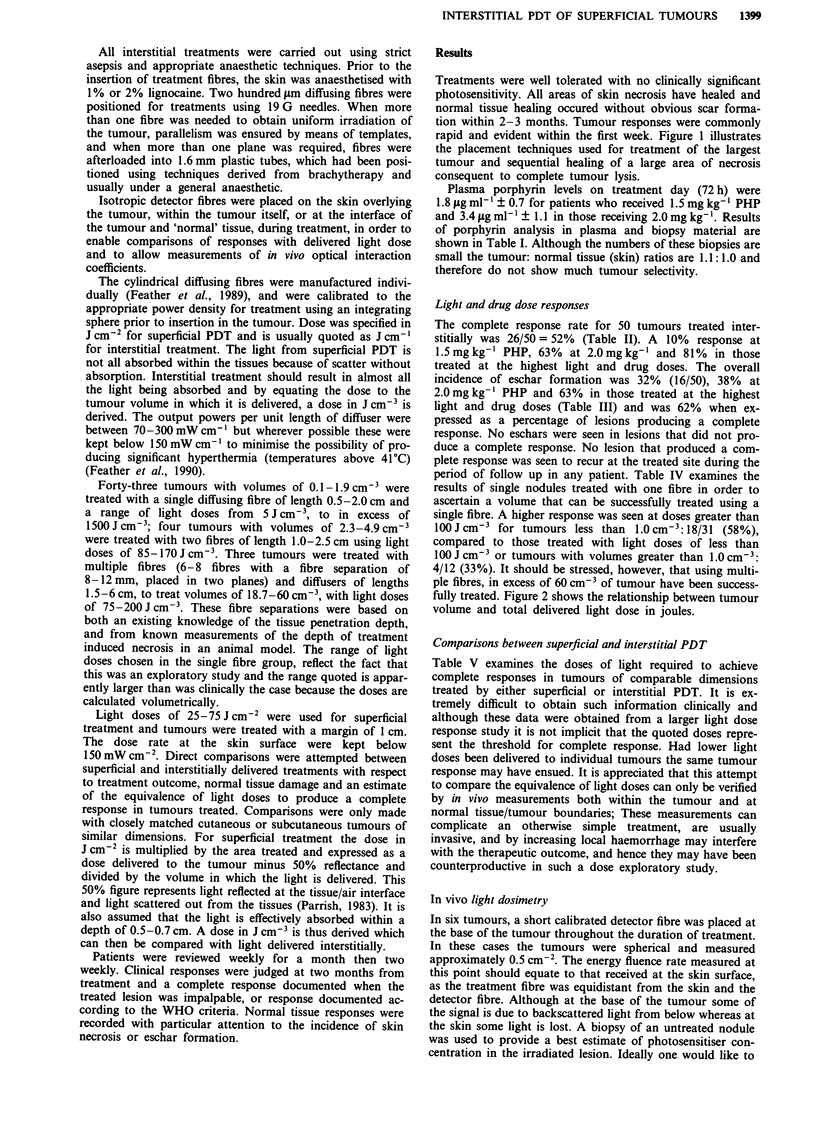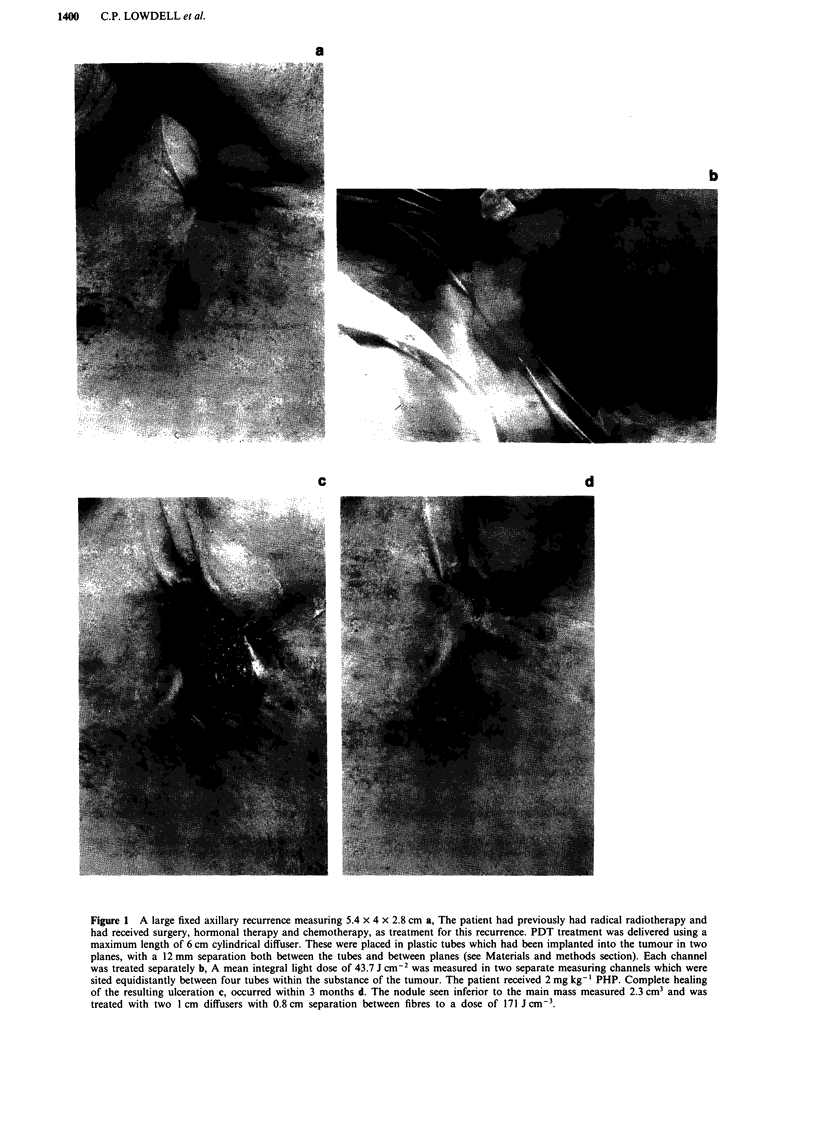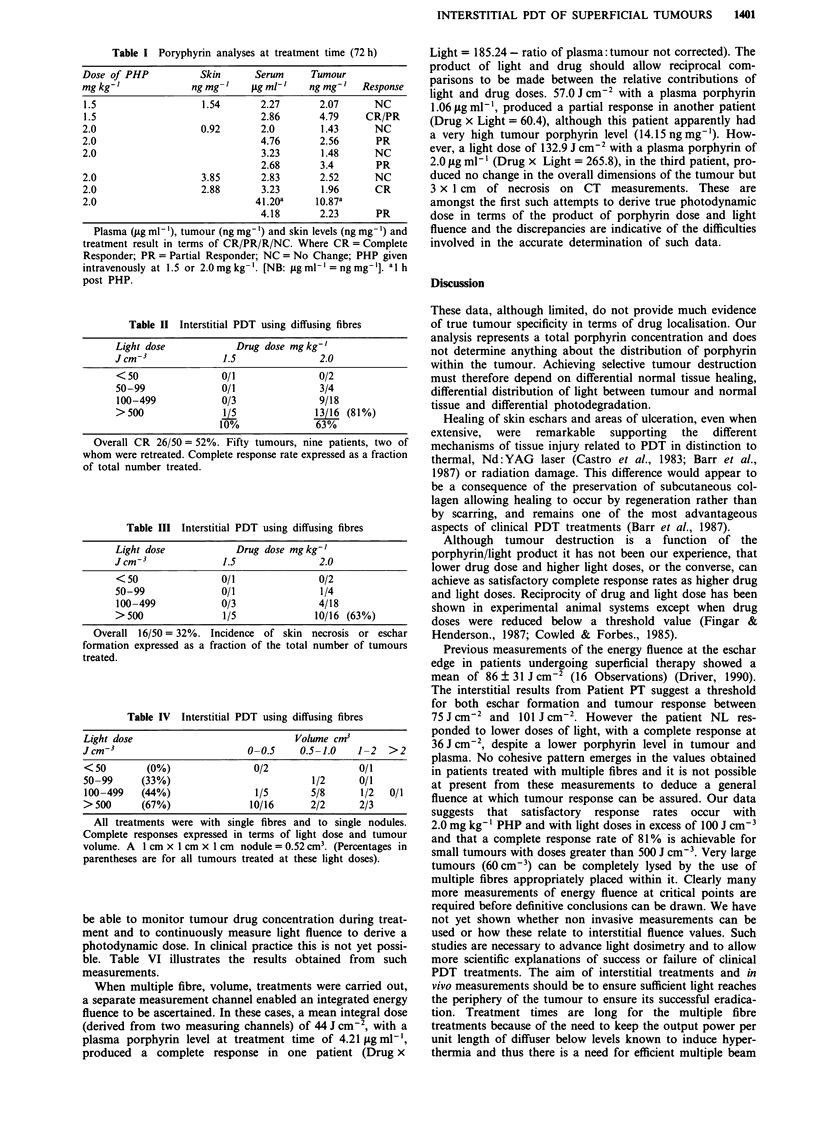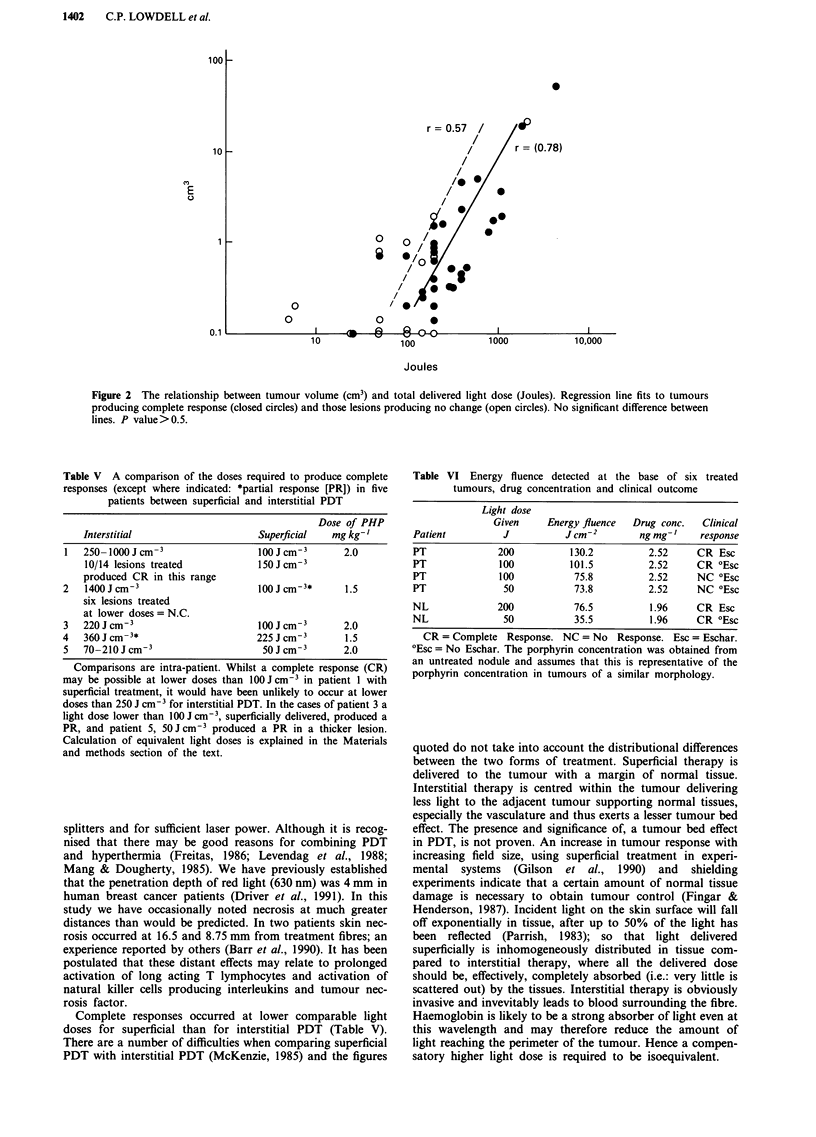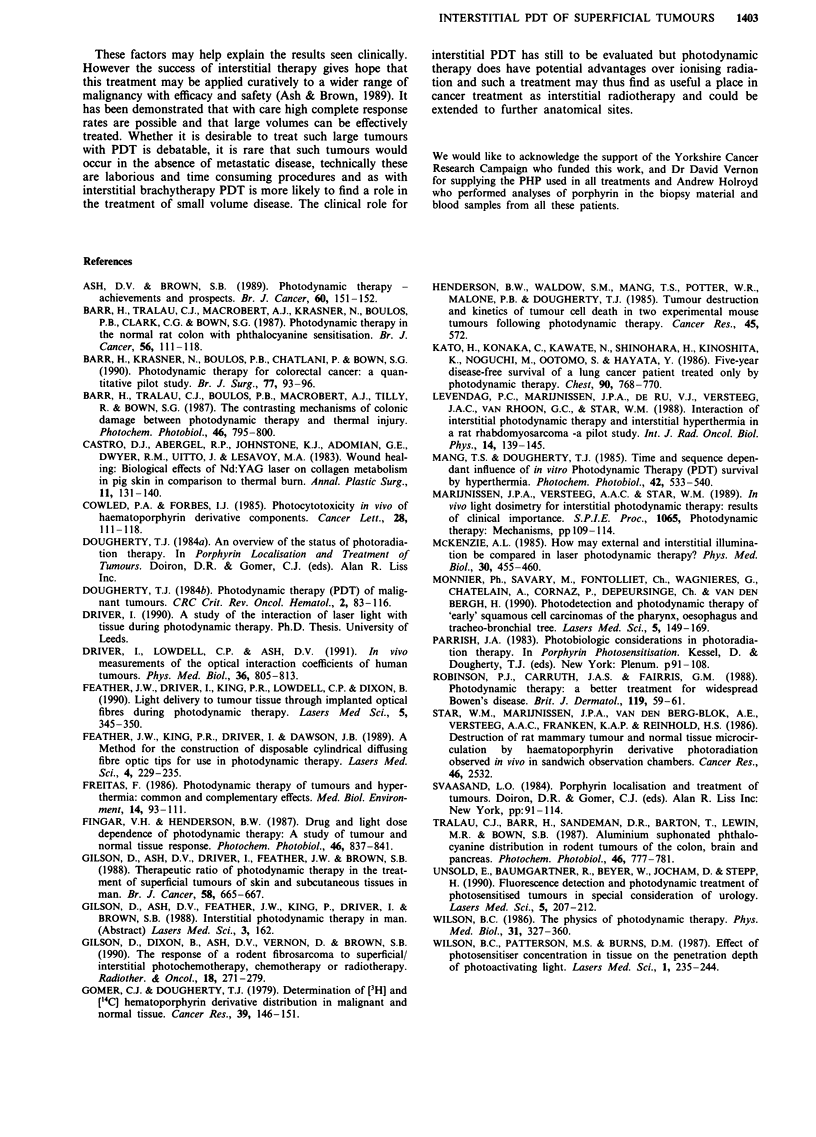# Interstitial photodynamic therapy. Clinical experience with diffusing fibres in the treatment of cutaneous and subcutaneous tumours.

**DOI:** 10.1038/bjc.1993.259

**Published:** 1993-06

**Authors:** C. P. Lowdell, D. V. Ash, I. Driver, S. B. Brown

**Affiliations:** Department of Radiotherapy, University of Leeds, Cookridge Hospital, UK.

## Abstract

**Images:**


Br. J. Cancer (1993), 67, 1398–1403 © Macmillan Press Ltd., 1993

### Interstitial photodynamic therapy. Clinical experience with diffusing fibres in the treatment of cutaneous and subcutaneous tumours

C.P. Lowdell[1], D.V. Ash[1], I. Driver[2] & S.B. Brown[3]

[1]*Department of Radiotherapy, University of Leeds, Cookridge Hospital, Leeds LS16 6QB;* [2]*Department of Medical Physics, University of Leeds, General Infirmary at Leeds, Great George Street, Leeds LS1 3EX;* [3]*Department of Biochemistry and Molecular Biology, University of Leeds, Leeds LS2 9JT, UK.*

**Summary** Interstitial photodynamic therapy has a number of potential advantages over superficial treatment. We have treated 50 subcutaneous and cutaneous tumours interstitially, in nine patients. An additional 22 tumours in the same patients, were treated by superficial PDT. Patients received 1.5–2.0 mg $kg^{-1}$ of polyhaematoporphyrin and 72 h later underwent treatment using a copper vapour dye laser producing red light at 630 nm.

All interstitial treatments were delivered using cylindrical diffusing fibres and a wide range of light doses (5-1500 J $cm^{-3}$). The complete response rate for all tumours treated interstitially was 52%, rising to 81% in those patients who received 2.0 mg $kg^{-1}$ PHP and light doses in excess of 500 J $cm^{-3}$. The overall incidence of skin necrosis was 32% and was 79% in those treated with light doses of greater than 500 J $cm^{-3}$. The incidence of skin necrosis with interstitial PDT is lower than that seen with superficial photodynamic therapy but higher volumetric light doses are required to produce tumour complete responses. All treatments were well tolerated and volumes of tumour up to 60 $cm^3$ were successfully treated. The penetration depth of 630 nm light in human breast cancer tissue was determined as 4 mm. Little true tumour tissue selectivity was detected by analysis of porphyrin levels in biopsy material.

Photodynamic therapy (PDT) generally involves the systemic administration of a photosensitiser that is retained with some selectivity in tumour tissue when compared with the normal tissue from which the tumour arose (Gomer *et al.*, 1979; Tralau *et al.*, 1987). The drug is then photoactivated by light of the appropriate wavelength and this leads to localised tumour necrosis which is primarily consequent to vascular collapse (Henderson *et al.*, 1985; Star *et al.*, 1986). It can be applied safely after other modalities of treatment such as radiation or chemotherapy (Dougherty, 1984; Gilson *et al.*, 1988). The results of clinical studies demonstrate that superficial therapy can be very effective for small superficial tumours (Dougherty, 1984; Gilson *et al.*, 1988; Kato *et al.*, 1986; Parrish, 1983; Robinson *et al.*, 1988; Unsold *et al.*, 1990) and that interstitial therapy shows promise for the treatment of small tumours, but should also allow treatment of non-superficial tumours (Barr *et al.*, 1990; Monnier *et al.*, 1990).

One of the major disadvantages of current PDT, especially of relevance to superficial treatment, relates to the poor penetration of red light at 630 nm. Previous studies have shown that the optical penetration depth (1/e or 37% level) in tumours, ranges from 1–3 mm (Svaasand, 1984), 2.7–4.5 mm (Wilson, 1986) and more recently *in vivo* measurements in human breast tumours 2.9–4.7 (Driver *et al.*, 1991). Photoactivation can occur at greater depths than this and effective penetration of up to 1 cm can be achieved (Dougherty, 1984; Gilson *et al.*, 1988). Newer drugs activated at longer wavelengths offer the potential to improve the penetration depths by a few millimetres, but are not likely to extend it much beyond this.

Interstitial therapy, using multiple fibres, should enable tumours of much larger volumes to be effectively treated and by delivering the light to the point of interest, limit the volume of normal tissue irradiated. Also, by reducing the incident light dose to the skin surface, it should decrease the incidence of skin necrosis, which is a feature of superficial therapy. In animal tumour systems interstitial PDT has been shown to be superior to superficial PDT (Marijnissen *et al.*, 1989). We have previously shown that plane cut fibres are unsatisfactory for interstitial PDT (Gilson *et al.*, 1988), because of thermal effects (Feather *et al.*, 1990) at the fibre tip and that diffusing fibres are the fibres of choice for interstitial PDT.

The aims of this study were to investigate interstitial PDT using cylindrical diffusing fibres. To identify response criteria in respect of light and drug doses for tumour and normal tissue effects, determine the volume of tumour that could be successfully treated interstitially and to compare interstitial treatment with superficial therapy in relation to its efficacy and examine the equivalence of light dose from these two methods of light delivery.

### Patients and methods

Between November 1988 and January 1990 nine patients aged 46–79 (mean 66) were treated, two of whom were re-treated, photodynamic therapy. All had received prior treatment with radiotherapy and often chemotherapy. Five had adenocarcinomas of the breast, three squamous carcinoma of the lung and one squamous carcinoma of the pinna. All had either cutaneous or subcutaneous metastases or a mixture of both. Fifty tumours were treated using purely interstitial techniques with cylindrical diffusing fibres. Twenty additional tumours in these same patients were treated by superficial photodynamic therapy and the response data accumulated were used to compare light dose equivalence with matched tumours treated by interstitial PDT. All tumours were carefully measured in three dimensions using vernier calipers and allowed tumour volumes to be calculated.

Patients were all treated as in-patients. All gave informed consent and received either 1.5 mg $kg^{-1}$ or 2.0 mg $kg^{-1}$ polyhaematoporphyrin (PHP) intravenously. PHP is a material apparently identical to Photofrin II in chemical and biological tests and is produced in our laboratories. Seventy-two hours later the tumours were irradiated with 630 nm light from a copper vapour dye laser. Patients were carefully counselled with respect to permissable levels of exposure to light.

Ten tumour and four skin biopsies were performed and assayed for porphyrin levels with corresponding serum samples by the Department of Biochemistry, University of Leeds, using spectrofluorimetry.

Correspondence: C.P. Lowdell, Department of Radiotherapy, Charing Cross Hospital, Fulham Palace Road, London W6 8RF, UK.

All interstitial treatments were carried out using strict asepsis and appropriate anaesthetic techniques. Prior to the insertion of treatment fibres, the skin was anaesthetised with 1% or 2% lignocaine. Two hundred μm diffusing fibres were positioned for treatments using 19 G needles. When more than one fibre was needed to obtain uniform irradiation of the tumour, parallelism was ensured by means of templates, and when more than one plane was required, fibres were afterloaded into 1.6 mm plastic tubes, which had been positioned using techniques derived from brachytherapy and usually under a general anaesthetic.

Isotropic detector fibres were placed on the skin overlying the tumour, within the tumour itself, or at the interface of the tumour and 'normal' tissue, during treatment, in order to enable comparisons of responses with delivered light dose and to allow measurements of *in vivo* optical interaction coefficients.

The cylindrical diffusing fibres were manufactured individually (Feather *et al.*, 1989), and were calibrated to the appropriate power density for treatment using an integrating sphere prior to insertion in the tumour. Dose was specified in $J\,cm^{-2}$ for superficial PDT and is usually quoted as $J\,cm^{-1}$ for interstitial treatment. The light from superficial PDT is not all absorbed within the tissues because of scatter without absorption. Interstitial treatment should result in almost all the light being absorbed and by equating the dose to the tumour volume in which it is delivered, a dose in $J\,cm^{-3}$ is derived. The output powers per unit length of diffuser were between 70–300 $mW\,cm^{-1}$ but wherever possible these were kept below 150 $mW\,cm^{-1}$ to minimise the possibility of producing significant hyperthermia (temperatures above 41°C) (Feather *et al.*, 1990).

Forty-three tumours with volumes of 0.1–1.9 $cm^{-3}$ were treated with a single diffusing fibre of length 0.5–2.0 cm and a range of light doses from 5 $J\,cm^{-3}$, to in excess of 1500 $J\,cm^{-3}$; four tumours with volumes of 2.3–4.9 $cm^{-3}$ were treated with two fibres of length 1.0–2.5 cm using light doses of 85–170 $J\,cm^{-3}$. Three tumours were treated with multiple fibres (6–8 fibres with a fibre separation of 8–12 mm, placed in two planes) and diffusers of lengths 1.5–6 cm, to treat volumes of 18.7–60 $cm^{-3}$, with light doses of 75–200 $J\,cm^{-3}$. These fibre separations were based on both an existing knowledge of the tissue penetration depth, and from known measurements of the depth of treatment induced necrosis in an animal model. The range of light doses chosen in the single fibre group, reflect the fact that this was an exploratory study and the range quoted is apparently larger than was clinically the case because the doses are calculated volumetrically.

Light doses of 25–75 $J\,cm^{-2}$ were used for superficial treatment and tumours were treated with a margin of 1 cm. The dose rate at the skin surface were kept below 150 $mW\,cm^{-2}$. Direct comparisons were attempted between superficial and interstitially delivered treatments with respect to treatment outcome, normal tissue damage and an estimate of the equivalence of light doses to produce a complete response in tumours treated. Comparisons were only made with closely matched cutaneous or subcutaneous tumours of similar dimensions. For superficial treatment the dose in $J\,cm^{-2}$ is multiplied by the area treated and expressed as a dose delivered to the tumour minus 50% reflectance and divided by the volume in which the light is delivered. This 50% figure represents light reflected at the tissue/air interface and light scattered out from the tissues (Parrish, 1983). It is also assumed that the light is effectively absorbed within a depth of 0.5–0.7 cm. A dose in $J\,cm^{-3}$ is thus derived which can then be compared with light delivered interstitially.

Patients were reviewed weekly for a month then two weekly. Clinical responses were judged at two months from treatment and a complete response documented when the treated lesion was impalpable, or response documented according to the WHO criteria. Normal tissue responses were recorded with particular attention to the incidence of skin necrosis or eschar formation.

### Results

Treatments were well tolerated with no clinically significant photosensitivity. All areas of skin necrosis have healed and normal tissue healing occured without obvious scar formation within 2–3 months. Tumour responses were commonly rapid and evident within the first week. Figure 1 illustrates the placement techniques used for treatment of the largest tumour and sequential healing of a large area of necrosis consequent to complete tumour lysis.

Plasma porphyrin levels on treatment day (72 h) were 1.8 $\mu g\,ml^{-1} \pm 0.7$ for patients who received 1.5 $mg\,kg^{-1}$ PHP and 3.4 $\mu g\,ml^{-1} \pm 1.1$ in those receiving 2.0 $mg\,kg^{-1}$. Results of porphyrin analysis in plasma and biopsy material are shown in Table I. Although the numbers of these biopsies are small the tumour: normal tissue (skin) ratios are 1.1:1.0 and therefore do not show much tumour selectivity.

### *Light and drug dose responses*

The complete response rate for 50 tumours treated interstitially was 26/50 = 52% (Table II). A 10% response at 1.5 $mg\,kg^{-1}$ PHP, 63% at 2.0 $mg\,kg^{-1}$ and 81% in those treated at the highest light and drug doses. The overall incidence of eschar formation was 32% (16/50), 38% at 2.0 $mg\,kg^{-1}$ PHP and 63% in those treated at the highest light and drug doses (Table III) and was 62% when expressed as a percentage of lesions producing a complete response. No eschars were seen in lesions that did not produce a complete response. No lesion that produced a complete response was seen to recur at the treated site during the period of follow up in any patient. Table IV examines the results of single nodules treated with one fibre in order to ascertain a volume that can be successfully treated using a single fibre. A higher response was seen at doses greater than 100 $J\,cm^{-3}$ for tumours less than 1.0 $cm^{-3}$: 18/31 (58%), compared to those treated with light doses of less than 100 $J\,cm^{-3}$ or tumours with volumes greater than 1.0 $cm^{-3}$: 4/12 (33%). It should be stressed, however, that using multiple fibres, in excess of 60 $cm^{-3}$ of tumour have been successfully treated. Figure 2 shows the relationship between tumour volume and total delivered light dose in joules.

### *Comparisons between superficial and interstitial PDT*

Table V examines the doses of light required to achieve complete responses in tumours of comparable dimensions treated by either superficial or interstitial PDT. It is extremely difficult to obtain such information clinically and although these data were obtained from a larger light dose response study it is not implicit that the quoted doses represent the threshold for complete response. Had lower light doses been delivered to individual tumours the same tumour response may have ensued. It is appreciated that this attempt to compare the equivalence of light doses can only be verified by *in vivo* measurements both within the tumour and at normal tissue/tumour boundaries; These measurements can complicate an otherwise simple treatment, are usually invasive, and by increasing local haemorrhage may interfere with the therapeutic outcome, and hence they may have been counterproductive in such a dose exploratory study.

### In vivo *light dosimetry*

In six tumours, a short calibrated detector fibre was placed at the base of the tumour throughout the duration of treatment. In these cases the tumours were spherical and measured approximately 0.5 $cm^{-2}$. The energy fluence rate measured at this point should equate to that received at the skin surface, as the treatment fibre was equidistant from the skin and the detector fibre. Although at the base of the tumour some of the signal is due to backscattered light from below whereas at the skin some light is lost. A biopsy of an untreated nodule was used to provide a best estimate of photosensitiser concentration in the irradiated lesion. Ideally one would like to

**Figure 1** A large fixed axillary recurrence measuring 5.4 × 4 × 2.8 cm **a**, The patient had previously had radical radiotherapy and had received surgery, hormonal therapy and chemotherapy, as treatment for this recurrence. PDT treatment was delivered using a maximum length of 6 cm cylindrical diffuser. These were placed in plastic tubes which had been implanted into the tumour in two planes, with a 12 mm separation both between the tubes and between planes (see Materials and methods section). Each channel was treated separately **b**, A mean integral light dose of 43.7 J $cm^{-2}$ was measured in two separate measuring channels which were sited equidistantly between four tubes within the substance of the tumour. The patient received 2 mg $kg^{-1}$ PHP. Complete healing of the resulting ulceration **c**, occurred within 3 months **d**. The nodule seen inferior to the main mass measured 2.3 $cm^3$ and was treated with two 1 cm diffusers with 0.8 cm separation between fibres to a dose of 171 J $cm^{-3}$.

**Table I** Poryphyrin analyses at treatment time (72 h)

| *Dose of PHP mg kg$^{-1}$* | *Skin ng mg$^{-1}$* | *Serum µg ml$^{-1}$* | *Tumour ng mg$^{-1}$* | *Response* |
|---|---|---|---|---|
| 1.5 | 1.54 | 2.27 | 2.07 | NC |
| 1.5 | | 2.86 | 4.79 | CR/PR |
| 2.0 | 0.92 | 2.0 | 1.43 | NC |
| 2.0 | | 4.76 | 2.56 | PR |
| 2.0 | | 3.23 | 1.48 | NC |
| | | 2.68 | 3.4 | PR |
| 2.0 | 3.85 | 2.83 | 2.52 | NC |
| 2.0 | 2.88 | 3.23 | 1.96 | CR |
| 2.0 | | 41.20[a] | 10.87[a] | |
| | | 4.18 | 2.23 | PR |

Plasma (µg ml$^{-1}$), tumour (ng mg$^{-1}$) and skin levels (ng mg$^{-1}$) and treatment result in terms of CR/PR/R/NC. Where CR = Complete Responder; PR = Partial Responder; NC = No Change; PHP given intravenously at 1.5 or 2.0 mg kg$^{-1}$. [NB: µg ml$^{-1}$ = ng mg$^{-1}$]. [a]1 h post PHP.

**Table II** Interstitial PDT using diffusing fibres

| *Light dose J cm$^{-3}$* | *Drug dose mg kg$^{-1}$ 1.5* | *2.0* |
|---|---|---|
| <50 | 0/1 | 0/2 |
| 50–99 | 0/1 | 3/4 |
| 100–499 | 0/3 | 9/18 |
| >500 | 1/5 | 13/16 (81%) |
| | 10% | 63% |

Overall CR 26/50 = 52%. Fifty tumours, nine patients, two of whom were retreated. Complete response rate expressed as a fraction of total number treated.

**Table III** Interstitial PDT using diffusing fibres

| *Light dose J cm$^{-3}$* | *Drug dose mg kg$^{-1}$ 1.5* | *2.0* |
|---|---|---|
| <50 | 0/1 | 0/2 |
| 50–99 | 0/1 | 1/4 |
| 100–499 | 0/3 | 4/18 |
| >500 | 1/5 | 10/16 (63%) |

Overall 16/50 = 32%. Incidence of skin necrosis or eschar formation expressed as a fraction of the total number of tumours treated.

**Table IV** Interstitial PDT using diffusing fibres

| *Light dose J cm$^{-3}$* | | *Volume cm$^3$ 0–0.5* | *0.5–1.0* | *1–2* | *>2* |
|---|---|---|---|---|---|
| <50 | (0%) | 0/2 | | 0/1 | |
| 50–99 | (33%) | | 1/2 | 0/1 | |
| 100–499 | (44%) | 1/5 | 5/8 | 1/2 | 0/1 |
| >500 | (67%) | 10/16 | 2/2 | 2/3 | |

All treatments were with single fibres and to single nodules. Complete responses expressed in terms of light dose and tumour volume. A 1 cm × 1 cm × 1 cm nodule = 0.52 cm$^3$. (Percentages in parentheses are for all tumours treated at these light doses).

be able to monitor tumour drug concentration during treatment and to continuously measure light fluence to derive a photodynamic dose. In clinical practice this is not yet possible. Table VI illustrates the results obtained from such measurements.

When multiple fibre, volume, treatments were carried out, a separate measurement channel enabled an integrated energy fluence to be ascertained. In these cases, a mean integral dose (derived from two measuring channels) of 44 J cm$^{-2}$, with a plasma porphyrin level at treatment time of 4.21 µg ml$^{-1}$, produced a complete response in one patient (Drug × Light = 185.24 – ratio of plasma:tumour not corrected). The product of light and drug should allow reciprocal comparisons to be made between the relative contributions of light and drug doses. 57.0 J cm$^{-2}$ with a plasma porphyrin 1.06 µg ml$^{-1}$, produced a partial response in another patient (Drug × Light = 60.4), although this patient apparently had a very high tumour porphyrin level (14.15 ng mg$^{-1}$). However, a light dose of 132.9 J cm$^{-2}$ with a plasma porphyrin of 2.0 µg ml$^{-1}$ (Drug × Light = 265.8), in the third patient, produced no change in the overall dimensions of the tumour but 3 × 1 cm of necrosis on CT measurements. These are amongst the first such attempts to derive true photodynamic dose in terms of the product of porphyrin dose and light fluence and the discrepancies are indicative of the difficulties involved in the accurate determination of such data.

### Discussion

These data, although limited, do not provide much evidence of true tumour specificity in terms of drug localisation. Our analysis represents a total porphyrin concentration and does not determine anything about the distribution of porphyrin within the tumour. Achieving selective tumour destruction must therefore depend on differential normal tissue healing, differential distribution of light between tumour and normal tissue and differential photodegradation.

Healing of skin eschars and areas of ulceration, even when extensive, were remarkable supporting the different mechanisms of tissue injury related to PDT in distinction to thermal, Nd:YAG laser (Castro *et al.*, 1983; Barr *et al.*, 1987) or radiation damage. This difference would appear to be a consequence of the preservation of subcutaneous collagen allowing healing to occur by regeneration rather than by scarring, and remains one of the most advantageous aspects of clinical PDT treatments (Barr *et al.*, 1987).

Although tumour destruction is a function of the porphyrin/light product it has not been our experience, that lower drug dose and higher light doses, or the converse, can achieve as satisfactory complete response rates as higher drug and light doses. Reciprocity of drug and light dose has been shown in experimental animal systems except when drug doses were reduced below a threshold value (Fingar & Henderson., 1987; Cowled & Forbes., 1985).

Previous measurements of the energy fluence at the eschar edge in patients undergoing superficial therapy showed a mean of 86 ± 31 J cm$^{-2}$ (16 Observations) (Driver, 1990). The interstitial results from Patient PT suggest a threshold for both eschar formation and tumour response between 75 J cm$^{-2}$ and 101 J cm$^{-2}$. However the patient NL responded to lower doses of light, with a complete response at 36 J cm$^{-2}$, despite a lower porphyrin level in tumour and plasma. No cohesive pattern emerges in the values obtained in patients treated with multiple fibres and it is not possible at present from these measurements to deduce a general fluence at which tumour response can be assured. Our data suggests that satisfactory response rates occur with 2.0 mg kg$^{-1}$ PHP and with light doses in excess of 100 J cm$^{-3}$ and that a complete response rate of 81% is achievable for small tumours with doses greater than 500 J cm$^{-3}$. Very large tumours (60 cm$^{-3}$) can be completely lysed by the use of multiple fibres appropriately placed within it. Clearly many more measurements of energy fluence at critical points are required before definitive conclusions can be drawn. We have not yet shown whether non invasive measurements can be used or how these relate to interstitial fluence values. Such studies are necessary to advance light dosimetry and to allow more scientific explanations of success or failure of clinical PDT treatments. The aim of interstitial treatments and *in vivo* measurements should be to ensure sufficient light reaches the periphery of the tumour to ensure its successful eradication. Treatment times are long for the multiple fibre treatments because of the need to keep the output power per unit length of diffuser below levels known to induce hyperthermia and thus there is a need for efficient multiple beam

**Figure 2** The relationship between tumour volume ($cm^3$) and total delivered light dose (Joules). Regression line fits to tumours producing complete response (closed circles) and those lesions producing no change (open circles). No significant difference between lines. $P$ value$>0.5$.

**Table V** A comparison of the doses required to produce complete responses (except where indicated: *partial response [PR]) in five patients between superficial and interstitial PDT

| | *Interstitial* | *Superficial* | *Dose of PHP mg kg⁻¹* |
|---|---|---|---|
| 1 | 250–1000 J $cm^{-3}$ 10/14 lesions treated produced CR in this range | 100 J $cm^{-3}$ 150 J $cm^{-3}$ | 2.0 |
| 2 | 1400 J $cm^{-3}$ six lesions treated at lower doses = N.C. | 100 J $cm^{-3}$* | 1.5 |
| 3 | 220 J $cm^{-3}$ | 100 J $cm^{-3}$ | 2.0 |
| 4 | 360 J $cm^{-3}$* | 225 J $cm^{-3}$ | 1.5 |
| 5 | 70–210 J $cm^{-3}$ | 50 J $cm^{-3}$ | 2.0 |

Comparisons are intra-patient. Whilst a complete response (CR) may be possible at lower doses than 100 J $cm^{-3}$ in patient 1 with superficial treatment, it would have been unlikely to occur at lower doses than 250 J $cm^{-3}$ for interstitial PDT. In the cases of patient 3 a light dose lower than 100 J $cm^{-3}$, superficially delivered, produced a PR, and patient 5, 50 J $cm^{-3}$ produced a PR in a thicker lesion. Calculation of equivalent light doses is explained in the Materials and methods section of the text.

**Table VI** Energy fluence detected at the base of six treated tumours, drug concentration and clinical outcome

| *Patient* | *Light dose Given J* | *Energy fluence J cm⁻²* | *Drug conc. ng mg⁻¹* | *Clinical response* |
|---|---|---|---|---|
| PT | 200 | 130.2 | 2.52 | CR Esc |
| PT | 100 | 101.5 | 2.52 | CR °Esc |
| PT | 100 | 75.8 | 2.52 | NC °Esc |
| PT | 50 | 73.8 | 2.52 | NC °Esc |
| NL | 200 | 76.5 | 1.96 | CR Esc |
| NL | 50 | 35.5 | 1.96 | CR °Esc |

CR = Complete Response. NC = No Response. Esc = Eschar. °Esc = No Eschar. The porphyrin concentration was obtained from an untreated nodule and assumes that this is representative of the porphyrin concentration in tumours of a similar morphology.

splitters and for sufficient laser power. Although it is recognised that there may be good reasons for combining PDT and hyperthermia (Freitas, 1986; Levendag *et al.*, 1988; Mang & Dougherty, 1985). We have previously established that the penetration depth of red light (630 nm) was 4 mm in human breast cancer patients (Driver *et al.*, 1991). In this study we have occasionally noted necrosis at much greater distances than would be predicted. In two patients skin necrosis occurred at 16.5 and 8.75 mm from treatment fibres; an experience reported by others (Barr *et al.*, 1990). It has been postulated that these distant effects may relate to prolonged activation of long acting T lymphocytes and activation of natural killer cells producing interleukins and tumour necrosis factor.

Complete responses occurred at lower comparable light doses for superficial than for interstitial PDT (Table V). There are a number of difficulties when comparing superficial PDT with interstitial PDT (McKenzie, 1985) and the figures quoted do not take into account the distributional differences between the two forms of treatment. Superficial therapy is delivered to the tumour with a margin of normal tissue. Interstitial therapy is centred within the tumour delivering less light to the adjacent tumour supporting normal tissues, especially the vasculature and thus exerts a lesser tumour bed effect. The presence and significance of, a tumour bed effect in PDT, is not proven. An increase in tumour response with increasing field size, using superficial treatment in experimental systems (Gilson *et al.*, 1990) and shielding experiments indicate that a certain amount of normal tissue damage is necessary to obtain tumour control (Fingar & Henderson, 1987). Incident light on the skin surface will fall off exponentially in tissue, after up to 50% of the light has been reflected (Parrish, 1983); so that light delivered superficially is inhomogeneously distributed in tissue compared to interstitial therapy, where all the delivered dose should be, effectively, completely absorbed (i.e.: very little is scattered out) by the tissues. Interstitial therapy is obviously invasive and invevitably leads to blood surrounding the fibre. Haemoglobin is likely to be a strong absorber of light even at this wavelength and may therefore reduce the amount of light reaching the perimeter of the tumour. Hence a compensatory higher light dose is required to be isoequivalent.

These factors may help explain the results seen clinically. However the success of interstitial therapy gives hope that this treatment may be applied curatively to a wider range of malignancy with efficacy and safety (Ash & Brown, 1989). It has been demonstrated that with care high complete response rates are possible and that large volumes can be effectively treated. Whether it is desirable to treat such large tumours with PDT is debatable, it is rare that such tumours would occur in the absence of metastatic disease, technically these are laborious and time consuming procedures and as with interstitial brachytherapy PDT is more likely to find a role in the treatment of small volume disease. The clinical role for interstitial PDT has still to be evaluated but photodynamic therapy does have potential advantages over ionising radiation and such a treatment may thus find as useful a place in cancer treatment as interstitial radiotherapy and could be extended to further anatomical sites.

We would like to acknowledge the support of the Yorkshire Cancer Research Campaign who funded this work, and Dr David Vernon for supplying the PHP used in all treatments and Andrew Holroyd who performed analyses of porphyrin in the biopsy material and blood samples from all these patients.

### References

ASH, D.V. & BROWN, S.B. (1989). Photodynamic therapy – achievements and prospects. *Br. J. Cancer*, **60**, 151–152.

BARR, H., TRALAU, C.J., MACROBERT, A.J., KRASNER, N., BOULOS, P.B., CLARK, C.G. & BOWN, S.G. (1987). Photodynamic therapy in the normal rat colon with phthalocyanine sensitisation. *Br. J. Cancer*, **56**, 111–118.

BARR, H., KRASNER, N., BOULOS, P.B., CHATLANI, P. & BOWN, S.G. (1990). Photodynamic therapy for colorectal cancer: a quantitative pilot study. *Br. J. Surg.*, **77**, 93–96.

BARR, H., TRALAU, C.J., BOULOS, P.B., MACROBERT, A.J., TILLY, R. & BOWN, S.G. (1987). The contrasting mechanisms of colonic damage between photodynamic therapy and thermal injury. *Photochem. Photobiol.*, **46**, 795–800.

CASTRO, D.J., ABERGEL, R.P., JOHNSTONE, K.J., ADOMIAN, G.E., DWYER, R.M., UITTO, J. & LESAVOY, M.A. (1983). Wound healing: Biological effects of Nd:YAG laser on collagen metabolism in pig skin in comparison to thermal burn. *Annal. Plastic Surg.*, **11**, 131–140.

COWLED, P.A. & FORBES, I.J. (1985). Photocytotoxicity *in vivo* of haematoporphyrin derivative components. *Cancer Lett.*, **28**, 111–118.

DOUGHERTY, T.J. (1984*a*). An overview of the status of photoradiation therapy. In *Porphyrin Localisation and Treatment of Tumours*. Doiron, D.R. & Gomer, C.J. (eds). Alan R. Liss Inc.

DOUGHERTY, T.J. (1984*b*). Photodynamic therapy (PDT) of malignant tumours. *CRC Crit. Rev. Oncol. Hematol.*, **2**, 83–116.

DRIVER, I. (1990). A study of the interaction of laser light with tissue during photodynamic therapy. Ph.D. Thesis. University of Leeds.

DRIVER, I., LOWDELL, C.P. & ASH, D.V. (1991). *In vivo* measurements of the optical interaction coefficients of human tumours. *Phys. Med. Biol.*, **36**, 805–813.

FEATHER, J.W., DRIVER, I., KING, P.R., LOWDELL, C.P. & DIXON, B. (1990). Light delivery to tumour tissue through implanted optical fibres during photodynamic therapy. *Lasers Med Sci.*, **5**, 345–350.

FEATHER, J.W., KING, P.R., DRIVER, I. & DAWSON, J.B. (1989). A Method for the construction of disposable cylindrical diffusing fibre optic tips for use in photodynamic therapy. *Lasers Med. Sci.*, **4**, 229–235.

FREITAS, F. (1986). Photodynamic therapy of tumours and hyperthermia: common and complementary effects. *Med. Biol. Environment*, **14**, 93–111.

FINGAR, V.H. & HENDERSON, B.W. (1987). Drug and light dose dependence of photodynamic therapy: A study of tumour and normal tissue response. *Photochem. Photobiol.*, **46**, 837–841.

GILSON, D., ASH, D.V., DRIVER, I., FEATHER, J.W. & BROWN, S.B. (1988). Therapeutic ratio of photodynamic therapy in the treatment of superficial tumours of skin and subcutaneous tissues in man. *Br. J. Cancer*, **58**, 665–667.

GILSON, D., ASH, D.V., FEATHER, J.W., KING, P., DRIVER, I. & BROWN, S.B. (1988). Interstitial photodynamic therapy in man. (Abstract) *Lasers Med. Sci.*, **3**, 162.

GILSON, D., DIXON, B., ASH, D.V., VERNON, D. & BROWN, S.B. (1990). The response of a rodent fibrosarcoma to superficial/interstitial photochemotherapy, chemotherapy or radiotherapy. *Radiother. & Oncol.*, **18**, 271–279.

GOMER, C.J. & DOUGHERTY, T.J. (1979). Determination of [$^3$H] and [$^{14}$C] hematoporphyrin derivative distribution in malignant and normal tissue. *Cancer Res.*, **39**, 146–151.

HENDERSON, B.W., WALDOW, S.M., MANG, T.S., POTTER, W.R., MALONE, P.B. & DOUGHERTY, T.J. (1985). Tumour destruction and kinetics of tumour cell death in two experimental mouse tumours following photodynamic therapy. *Cancer Res.*, **45**, 572.

KATO, H., KONAKA, C., KAWATE, N., SHINOHARA, H., KINOSHITA, K., NOGUCHI, M., OOTOMO, S. & HAYATA, Y. (1986). Five-year disease-free survival of a lung cancer patient treated only by photodynamic therapy. *Chest*, **90**, 768–770.

LEVENDAG, P.C., MARIJNISSEN, J.P.A., DE RU, V.J., VERSTEEG, J.A.C., VAN RHOON, G.C., & STAR, W.M. (1988). Interaction of interstitial photodynamic therapy and interstitial hyperthermia in a rat rhabdomyosarcoma -a pilot study. *Int. J. Rad. Oncol. Biol. Phys.*, **14**, 139–145.

MANG, T.S. & DOUGHERTY, T.J. (1985). Time and sequence dependant influence of *in vitro* Photodynamic Therapy (PDT) survival by hyperthermia. *Photochem. Photobiol.*, **42**, 533–540.

MARIJNISSEN, J.P.A., VERSTEEG, A.A.C. & STAR, W.M. (1989). *In vivo* light dosimetry for interstitial photodynamic therapy: results of clinical importance. *S.P.I.E. Proc.*, **1065**, Photodynamic therapy: Mechanisms, pp 109–114.

MCKENZIE, A.L. (1985). How may external and interstitial illumination be compared in laser photodynamic therapy? *Phys. Med. Biol.*, **30**, 455–460.

MONNIER, Ph., SAVARY, M., FONTOLLIET, Ch., WAGNIERES, G., CHATELAIN, A., CORNAZ, P., DEPEURSINGE, Ch. & VAN DEN BERGH, H. (1990). Photodetection and photodynamic therapy of 'early' squamous cell carcinomas of the pharynx, oesophagus and tracheo-bronchial tree. *Lasers Med. Sci.*, **5**, 149–169.

PARRISH, J.A. (1983). Photobiologic considerations in photoradiation therapy. In *Porphyrin Photosensitisation*. Kessel, D. & Dougherty, T.J. (eds). New York: Plenum. p91–108.

ROBINSON, P.J., CARRUTH, J.A.S. & FAIRRIS, G.M. (1988). Photodynamic therapy: a better treatment for widespread Bowen's disease. *Brit. J. Dermatol.*, **119**, 59–61.

STAR, W.M., MARIJNISSEN, J.P.A., VAN DEN BERG-BLOK, A.E., VERSTEEG, A.A.C., FRANKEN, K.A.P. & REINHOLD, H.S. (1986). Destruction of rat mammary tumour and normal tissue microcirculation by haematoporphyrin derivative photoradiation observed *in vivo* in sandwich observation chambers. *Cancer Res.*, **46**, 2532.

SVAASAND, L.O. (1984). Porphyrin localisation and treatment of tumours. Doiron, D.R. & Gomer, C.J. (eds). Alan R. Liss Inc: New York, pp:91–114.

TRALAU, C.J., BARR, H., SANDEMAN, D.R., BARTON, T., LEWIN, M.R. & BOWN, S.B. (1987). Aluminium suphonated phthalocyanine distribution in rodent tumours of the colon, brain and pancreas. *Photochem. Photobiol.*, **46**, 777–781.

UNSOLD, E., BAUMGARTNER, R., BEYER, W., JOCHAM, D. & STEPP, H. (1990). Fluorescence detection and photodynamic treatment of photosensitised tumours in special consideration of urology. *Lasers Med. Sci.*, **5**, 207–212.

WILSON, B.C. (1986). The physics of photodynamic therapy. *Phys. Med. Biol.*, **31**, 327–360.

WILSON, B.C., PATTERSON, M.S. & BURNS, D.M. (1987). Effect of photosensitiser concentration in tissue on the penetration depth of photoactivating light. *Lasers Med. Sci.*, **1**, 235–244.